# Ketogenic diet in cancer therapy

**DOI:** 10.18632/aging.101382

**Published:** 2018-02-11

**Authors:** Daniela D. Weber, Sepideh Aminazdeh-Gohari, Barbara Kofler

**Affiliations:** 1 Research Program for Receptor Biochemistry and Tumor Metabolism, University Hospital for Pediatrics of the Paracelsus Medical University, 5020, Salzburg, Austria

**Keywords:** tumor metabolism, ketogenic diet, adjuvant therapy

The Ketogenic Diet (KD), a high-fat/low-carbohydrate/adequate-protein diet, has recently been proposed as an adjuvant therapy in cancer treatment [1]. KDs target the Warburg effect, a biochemical phenomenon in which cancer cells predominantly utilize glycolysis instead of oxidative phosphorylation to produce ATP. Furthermore, some cancers lack the ability to metabolize ketone bodies, due to mitochondrial dysfunction and down-regulation of enzymes necessary for ketone utilization [2]. Thus, the rationale in providing a fat-rich, low-carbohydrate diet in cancer therapy is to reduce circulating glucose levels and induce ketosis such that cancer cells are starved of energy while normal cells adapt their metabolism to use ketone bodies and survive. Furthermore, by reducing blood glucose also levels of insulin and insulin-like growth factor, which are important drivers of cancer cell proliferation, drop.

**Figure 1:**
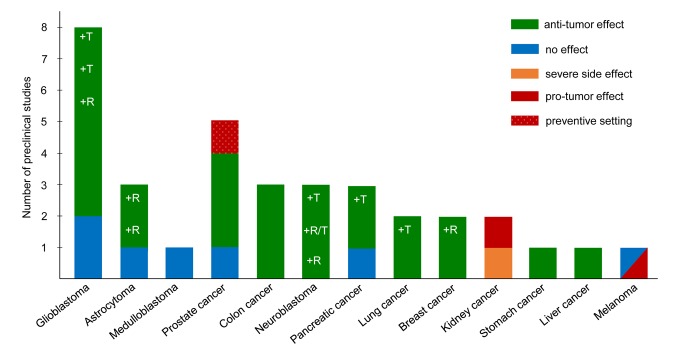
**Preclinical evidence indicating the effect of a KD on tumor growth and progression**. The bar chart shows the number of preclinical studies, which investigated the effect of a KD on different types of cancer. Colors of the bars represent the result of each study as indicated in the color key. Studies on KD and cancer were collected by a literature search covering through the end of 2017. R indicates studies with a calorie-restricted KD; T indicates use of a KD as an adjuvant therapy to classic therapy.

Numerous preclinical studies have provided evidence for an anti-tumor effect of KDs [1] (Figure 1). For example, our laboratory intensively studied the anti-tumor effect of KDs in combination with or without low-dose chemotherapy on neuroblastoma. We found that the growth of neuroblastoma xenografts was significantly reduced by a KD consisting of a 2:1 ratio of *fat* to *carbohydrate + protein* when combined with caloric restriction [2]. However, caloric restriction, despite its anti-tumor effect and potential to sensitize cancer cells to chemotherapy, would be contraindicated in a range of cancer patients, particularly those with cachexia. Thus, we further focused on optimizing the KD composition to address this issue. We found that an *ad libitum* KD (8:1) with a fat content of 25% medium-chain triglycerides and 75% long-chain triglycerides produced a stronger anti-tumor effect compared to a KD (8:1) with all long-chain triglycerides, and was as efficacious against neuroblastoma as the above-described KD (2:1) combined with caloric restriction [3]. These results stress the importance of an optimized KD composition to suppress tumor growth and to sensitize tumors to chemotherapy without requiring caloric restriction.

In addition to neuroblastoma, various researchers have investigated the efficacy of KDs as an adjuvant therapy for other types of cancer. The strongest evidence (> 3 studies) for a tumor-suppressing effect has been reported for glioblastoma, whereas little or no benefit was found for two other brain tumors (astrocytoma and medulloblastoma). Good evidence (2 - 3 studies) is available for prostate, colon, pancreatic and lung cancer [1]; neuroblastoma also falls into this category (Figure 1). Some of those studies report a tumor-suppressing effect of KD alone and/or in combination with classic therapy and/or caloric restriction. One study on prostate cancer applied the KD in a preventive, instead of a therapeutic, study setting. Only limited evidence (1 study) supports the anti-tumor effect of an unrestricted KD on breast, stomach, and liver cancer.

In contrast to the safe application of KDs reported in various cancer models, our research group recently reported that mice bearing renal cell carcinoma xenografts and with signs of Stauffer’s syndrome experienced dramatic weight loss and liver dysfunction when treated with a KD [4]. Another study investigating the effect of long-term KD treatment on kidney cancer described a pro-tumor effect of the KD in a rat model of tuberous sclerosis complex [5]. Most concerning is the observation that, in a mouse model of BRAF V600E-positive melanoma, tumor growth was significantly increased under the KD [6]. Moreover, the study also demonstrated that the ketone body acetoacetate stimulated the oncogenic signaling of the BRAF pathway. In contrast, the KD had no effect on the progression of NRAS Q61K-positive or wild-type melanoma xenografts [6]. Notwithstanding these observations, in a feasibility trial involving a limited number of patients with advanced malignancies, a patient with BRAF V600E-positive/BRAF-inhibitor resistant melanoma seemed to benefit from the KD [7].

Taken together, results from preclinical studies, albeit sometimes contradictory, tend to support an anti-tumor effect rather than a pro-tumor effect of the KD for most solid cancers. However, even though pro-tumor effects are rare, they cannot be ruled out per se. Most importantly, available preclinical evidence implies that the feasibility of a KD as an adjuvant cancer therapy strongly depends on the type of tumor and its genetic alterations.

To date, evidence from randomized controlled clinical trials is lacking, but needed, to answer the question of whether an adjuvant KD would benefit specific cancer patients. Human data pertaining to KDs and cancer are mostly based on single case reports and a smattering of preliminary clinical studies with small study cohorts, heterogenous study designs, poor compliance to the diet, noncomparable regimens, or without standardized dietary guidance. Even so, results of the first clinical studies support the hypothesis of an anti-tumor effect of KDs. For example, 10 of the 24 (42%) clinical studies included in a recent review [1] provide evidence for the anti-tumor effect of KDs, whereas seven (29%) showed no effect and only one study reported a pro-tumor effect of the KD. The currently available medical literature presents strong scientific evidence for the safe application of a KD only in patients with glioblastoma. However, a clear recommendation for adjuvant use of the KD in glioblastoma patients still requires results from ongoing randomized controlled clinical trials.

In conclusion, clinical application of KDs as an adjuvant therapy for cancer patients first requires that the KD be evaluated for its anti-tumor effect for each single type/genetic subtype of cancer in a preclinical setting, as the safety and efficacy of the KD strongly depend on the tumor entity and its genotype. Based on the results of rigorous preclinical and clinical studies performed thus far, the KD would appear to be a promising and powerful option for adjuvant therapy for a range of cancers. Cancer-specific recommendations await the findings of randomized controlled clinical trials.
